# Poult Enteritis and Mortality Syndrome in Turkey Poults: Causes, Diagnosis and Preventive Measures

**DOI:** 10.3390/ani11072063

**Published:** 2021-07-10

**Authors:** Awad A. Shehata, Shereen Basiouni, Reinhard Sting, Valerij Akimkin, Marc Hoferer, Hafez M. Hafez

**Affiliations:** 1 Birds and Rabbit Medicine Department, Faculty of Veterinary Medicine, University of Sadat City, Sadat City 32897, Egypt; 2 Research and Development Section, PerNaturam GmbH, 56290 Gödenroth, Germany; 3 Clinical Pathology Department, Faculty of Veterinary Medicine, Benha University, Benha 13518, Egypt; shereenbh@yahoo.com; 4 Chemisches und Veterinäruntersuchungsamt Stuttgart, 70736 Fellbach, Germany; reinhard.sting@cvuas.bwl.de (R.S.); Valerij.Akimkin@cvuas.bwl.de (V.A.); 5 Chemisches und Veterinäruntersuchungsamt Freiburg, 79108 Freiburg, Germany; Marc.Hoferer@cvuafr.bwl.de; 6 Institute of Poultry Diseases, Faculty of Veterinary Medicine, Free University of Berlin, 14163 Berlin, Germany

**Keywords:** turkeys, PEMS, astroviruses, coronaviruses, rotaviruses, probiotics, prebiotics, phytogenic substances

## Abstract

**Simple Summary:**

The poult enteritis and mortality syndrome (PEMS) causes severe economic losses in turkeys. Several agents were described to be associated with the PEMS; however, a specific etiological agent(s) has not been identified. The diagnosis of PEMS is still a huge challenge for several reasons: (1) no specific clinical signs or pathognomonic lesions, (2) isolation of some enteric viruses still difficult, (3) the pathogenicity of several enteric viruses in turkeys is not fully understood, (4) PEMS is an interaction between several known and might be unknown agents and (5) opportunistic microorganisms also have a role in the pathogenesis of PEMS. Both electron microscopy and molecular techniques can be used for diagnosis of PEMS and might help to discover unknown causes. Until now, no specific vaccines against enteric viruses associated with PEMS. However, biosecurity, maintaining a healthy gut and strengthening the immune system of turkey poults using probiotics, prebiotics and/or phytogenic substances are crucial factors to prevent and/or reduce losses of PEMS in turkeys. This review is a call for scientists to perform further research to investigate the real cause(s) of PEMS and to develop a preventive strategy against it.

**Abstract:**

Poult enteritis and mortality syndrome (PEMS) is one of the most significant problem affecting turkeys and continues to cause severe economic losses worldwide. Although the specific causes of PEMS remains unknown, this syndrome might involve an interaction between several causative agents such as enteropathogenic viruses (coronaviruses, rotavirus, astroviruses and adenoviruses) and bacteria and protozoa. Non-infectious causes such as feed and management are also interconnected factors. However, it is difficult to determine the specific cause of enteric disorders under field conditions. Additionally, similarities of clinical signs and lesions hamper the accurate diagnosis. The purpose of the present review is to discuss in detail the main viral possible causative agents of PEMS and challenges in diagnosis and control.

## 1. Introduction

Several challenges such as intense global competition between producing countries, permanent changes in social, political and consumer perceptions regarding food safety, animal welfare and environmental protection are influencing turkey production and health [1]. A healthy gastrointestinal tract (GIT) is the key toward successful poultry production. The fundamental role of turkey production is processing of feedstuffs into meat. The GIT is the most extensive surface in the body that is constantly exposed to various infections. Mechanical, chemical or biological disturbance of the digestive system usually negatively impacts this process, which is subsequently accompanied by high economic losses, serious problems are predominant in young birds [2]. In poultry, several viral infections either as monocausal, multicausal or viral infections accompanied by non-infectious factors are indeed causing high economic losses worldwide. These economic losses are due to high mortality rates and reduction of animal performance as a result of decreased weight gain, decreased egg production, decreased hatchability, increased medication costs and impaired feed conversion rates [3].

The poult enteritis complex (PEC) is common in turkeys and characterized by depression, enteritis, diarrhea, low feed conversion ratio and poor weight gain. It is a general term denoting all infectious intestinal diseases of young poults with uncertain etiology [4]. The PEC is caused by a group of multifactorial infectious and non-infectious agents with significant effect on turkeys less than six weeks old [5]. Several syndromes were reported as a part of PEC such as the poult enteritis and mortality syndrome (PEMS), maldigestion syndrome, runting stunting syndrome (RSS), poult malabsorption syndrome, spiking mortality of turkeys (SMT), poult enteritis syndrome (PES) in young turkeys between 1 day and 7 weeks and light turkey syndrome (LTS), a problem of lower body weight at market age turkeys [6,7]. In cases in which morbidity and mortality are high, the disease was classified as PEMS, which is an economically devastating condition [8].

Several causative agents are implicated as the main causes of PEMS such as enteroviruses (turkey corona virus (TCoV), turkey astroviruses (TAstV), reoviruses and adenoviruses)), and also bacteria such as *E. coli*, *Salmonella*, *Campylobacter*, *Clostridia*, *Cryptosporidium* and *Cochlosoma* [5,6,9,10,11,12,13,14,15]. The severity of the disease depends on the virulence of enteric viruses, coinfections, other interacting factors such as the age and immune status of the affected birds [16] and management, nutrition and hygienic measures (Table 1). Therefore, the true role of viruses in naturally occurring PEMS is difficult to assess as sole etiologies [17]. In this review, we will discuss the possible causes, diagnosis, and preventive measures of PEMS as a major challenge facing turkey production.

## 2. Causes of PEMS

### 2.1. Turkey Coronavirus

TCoV is known for about 70 years; it was first isolated in 1951 in the USA by Peterson and Hymas [18]. Later on, the virus was reported in several countries worldwide including Australia, Brazil, Italy, UK, France and Poland [19,20,21,22,23,24]. In Europe, TCoV was isolated for the first time in 2008 from turkey poults suffering from enteritis [21]. TCoV infections remain a leading cause of massive economic losses in young turkeys in many countries [25]. The virus belongs to the family Coronaviridae, which is classified into two subfamilies, namely, Letovirinae and Orthocoronavirinae. While the subfamily Letovirinae includes the genus *Alphaletovirus*, the subfamily Orthocoronaviridae contains four genera based on the phylogenetic analysis and genome structure: *Alphacoronavirus* (αCoV), *Betacoronavirus* (βCoV), *Gammacoronavirus* (γCoV) and *Deltacoronavirus* (δCoV), [26]. Both γCoV and δCoV infect birds, but some can also infect mammals [27,28]. The γCoV contains three subgenera, namely, *Igacovirus* and *Brangacovirus*, both identified in birds, and *Cegacovirus*, reported in mammals (beluga whale, SW1 virus) [26]. TCoV belongs to the genus γCoV and subgenus *Igacovirus*, which contains other avian coronaviruses (ACoVs) such as infectious bronchitis virus (IBV) and guinea fowl coronavirus (GfCoV). The virus is enveloped, containing single-stranded, positive-sense, non-segmented RNA of 28-kb [29,30].

Like other ACoVs, the genome of ACoV consists of 15 non-structural proteins, encoded by open reading frame (ORF) 1a/b at the 5’-end, and four structural proteins (spike (S), envelope (E), membrane (M) and nucleocapsid (N), encoded by other ORF at the 3´-end [21,31,32,33]. Generally, ACoVs have similar phylogenetic relationships and genomic structures and close nucleotide identities. The IBV, TCoV and GfCoV exhibited nucleotide identities of 90% for the replicase, E, M and N genes [28,29,34]. However, the S gene of ACoVs shares at most 36% identity [31,35]. Three distinct genetic groups of TCoV isolates in USA were identified, namely, in North Carolina isolates formed group I, Texas isolates formed group II, and Minnesota isolates formed group III, suggesting the endemic circulation of distinct TCoV genotypes in different geographic states [36]. Recombination in coronaviruses is common. Wang and others documented a recombination event between a chicken coronavirus and TCoV in China using viral metagenomic analysis [37]. Additionally, an atypical TCoV strain was isolated from duodenum of 5-week-old turkey poults suffering from acute enteritis in Poland. Molecular analysis revealed recombination between different γCoV genomic backbones, suggested potential transmission of coronaviruses between different bird species [32].

The TCoV is involved in the economically devastating PEMS, a multifactorial syndrome [10]. The natural host of TCoV is turkeys, as this virus did not cause a disease in chickens under experimental conditions [30]. TCoV causes high morbidity rates that may reach 100% and a sudden increase in mortality of 10–50% in turkeys during the first 4 weeks of age. Although TCoV is more common in young poults, exposure of older ages results in stunting with low mortality rates [38]. The natural route of infection of TCoV is orally by ingestion of contaminated fecal materials. It replicates in enterocytes at the apical portion of the intestinal villi in the jejunum and ileum and in the immune organs such as the bursa of Fabricius [39]. The virus was also detected in dendritic cells, monocytes and macrophages, highlighting its potential replication in antigen-presenting cells [40]. Movement of contaminated equipment, personal or vehicles and other birds probably spread the virus. The disease is more common and severe during summer months (May to August) with sporadic occurrence in autumn. Once turkeys are infected with the virus, they remain life-long shedders [11].

Post-mortem lesions are mainly found in the GIT and bursa of Fabricius. Pale duodenum and jejunum that are distended watery with gaseous contents were reported. In addition, ceca were distended and filled with watery contents. Atrophy of the bursa of Fabricius may also be observed. Microscopic lesions include villus atrophy, infiltration with mononuclear inflammatory cells in the lamina propria and decreased numbers of goblet cells on villous tips. Lymphoid atrophy of follicular cells of bursa of Fabricius and heterophilic infiltration are also reported [10,40,41]. Experimentally, TCoV shedding persists until 14-weeks post inoculation [42]. However, inoculation with the TCoV NC95 isolate was shed up to 7-weeks [43]. Additionally, latent infection with TCoV without clinical signs is reported [44].

### 2.2. Astrovirus

The TAstV was first reported in 1980 in turkey poults suffering from diarrhea and increased mortality in UK [45]. Since then, it has been documented worldwide [14,46,47,48]. TAstV infections are common in 4-week-old turkey poults as a coinfection in enteric disease. TAstVs, belonging to the family Astroviridiae, are non-enveloped, single stranded positive-sense RNA viruses with a genome size of 6.5–7.5 kb long. It contains three ORFs: ORF1a encodes the non-structural proteins serine-protease, ORFab encodes RNA-dependent RNA-polymerase and ORF2 encodes the structural proteins of the viral capsid [49]. Three astrovirus types, namely, TAstV-1 (7003 nt), TAstV-2 (7325 nt) and avian nephritis virus (ANV, 6927 nt) have been detected in commercial turkey flocks, with a prevalence of 100, 15.4 and 12.5%, respectively. The TAstV-2 has frequently been associated with PEC, PEMS and PES [5,6,50,51]. Additionally, TAstV-2 has been detected in apparently healthy flocks of turkeys [50,51]. The ANV is associated with nephritis and RSS in chicken and turkey flocks and other avian species [52].

The TAstVs replicates in the basal portion of the lamina and rarely in the crypts [53,54]. Oral inoculation of TAstV in one-day-old specific pathogen free (SPF) turkey poults decreased the absorption of D-xylose [55], resulting in maldigestion of disaccharides, malabsorption and consequent osmotic diarrhea [56]. It was also found that 24 h after experimental infections of one-day-old turkey poults, birds showed signs of intestinal infection including yellowish brown watery to foamy diarrhea, followed by emaciation and stunting growth [57,58]. Significant reduction of body weights as a result of decreased absorption of nutrients was also found [58]. The main pathological changes are mainly located also in the digestive tract and usually non-specific including dilated ceca with yellowish frothy contents, fluid distention and inflammation of intestines [57]. It was also suggested that TAstV virus might cause immunosuppression, hence the virus was detected in bursa and thymus [59].

### 2.3. Adenovirus

Adenoviruses, family Adenoviridae, are DNA viruses with an icosahedral capsid and a double-stranded, linear genome. Adenoviruses are described in many species of vertebrate animals, including mammals, birds, reptiles, amphibians and fish [60,61,62]. Three different genera namely, *Aviadenovirus*, *Siadenovirus* and *Atadenovirus* can infect poultry [63]. The *Aviadenovirus* comprises the fowl aviadenovirus (FAdV) and the turkey aviadenovirus (TAdV) species [63]. FAdVs are grouped into the five species (FAdV-A to FAdV-E), 12 serotypes (FAdV-1 to FAdV-8a and FAdV-8b to FAdV-11) and 12 genotypes. Three TAdV species, namely TAdV-B (type TAdV-1), TAdV-C (type TAdV-4) and TAdV-D (type TAdV-5) can infect turkeys [64,65,66], isolated from respiratory disease and PEMS. These viruses also cause inclusion body hepatitis in turkey poults and may be responsible for lower hatchability rates in breeder flocks [67,68]. Generally, further studies are required to understand the pathogenicity of aviadenoviruses in turkeys, hence all aviadenoviruses were identified within diseased turkey flocks in Germany, however, no apparent link between case history and type of isolate were identified [65].

Hemorrhagic enteritis (HE) has been reported worldwide, i.e., Canada, England, Germany, Australia, India, Japan, Israel and the USA [69]. Surprisingly, the HE virus does not replicate in intestinal epithelium; however, it replicates in the endothelial cells that causes vascular damage and ischemic necrosis of intestinal villi. HE virus causes severe immunosuppression in turkeys, which subsequently stimulate opportunistic bacteria [70]. The HE infection is common in 4–12-week-old turkey poults [69]. The main signs include depression, bloody droppings and sudden death [71]. The main post-mortem lesions are enlarged mottled spleen and distended and congested intestines, more prominent in the proximal small intestine. The intestine might be filled with bloody exudate [69,71]. Microscopic examination revealed characteristic lesions including hyperplasia of white pulp and lymphoid necrosis in the spleen at death [71]. Moreover, severely congested mucosa, degeneration and sloughing of the villus epithelium and hemorrhage at the villus tips were reported.

### 2.4. Rotaviruses

Rotaviruses, genus *Rotavirus*, family Reoviridae, have been associated with enteritis in mammalian and poultry species [72,73,74]. Rotaviruses are non-enveloped icosahedral particles and contain double stranded-RNA of 11 segments, which encode structural (VP1 to VP4, VP6 and VP7) and non-structural proteins (NSP1 to NSP6). According to VP6, rotaviruses are classified into 10 groups (RVA-RVJ) [75]. Turkey rotaviruses belong to group A rotaviruses [45], however, rotaviruses that are antigenically distinct (referred as rotavirus-like particles) from group A turkey rotaviruses were also detected in turkey poults [76]. The pathogenicity of rotaviruses in turkeys depends on several factors including the virulence of involved strains, coinfections with other pathogens and management [77]. The main clinical signs of rotaviruses infections are diarrhea, depression, high mortality rates, chronic runting and stunting.

### 2.5. Reovirus

Avian reoviruses have been associated with enteric disease, arthritis/tenosynovitis, respiratory distress, immunosuppression, poor feed conversion and malabsorption syndrome in poultry [78]. Avian reoviruses belong to family Orthoreoviridae, genus *Orthoreovirus*. These viruses are non-enveloped and have linear double stranded RNA with 10 segments. Fusogenic reovirus strains are characterized by the ability to fuse with infected cells and form multinucleated syncytia, affecting mammals, birds and reptiles, while non-fusogenic viruses are mainly infecting mammals [79]. Based on the molecular differences between avian reoviruses, species-specific reovirus types are being described, namely turkey reovirus for turkey, duck reovirus for duck, goose reovirus for goose and avian reovirus chickens [80]. Generally, young birds without maternal antibodies can be infected with reoviruses [81]. However, the course of infection depends on the age of birds and their sensitivity, pathogenicity of the reovirus strain, infectious dose, route of infection, presence of maternal antibodies and immune status.

Turkey reoviruses are associated with arthritis and PEMS in turkeys [82]. Turkey arthritis reovirus causes tenosynovitis in turkeys, leading to the reduction of performance due to mortality and low feed conversion ratio. Additionally, although reoviruses have been isolated from turkey poults suffering from enteritis disease, they have been isolated from apparently healthy birds [83]. Experimentally infected SPF poults showed mild clinical signs and exhibited no post-mortem lesions, highlighting that turkey reovirus might not be the primary cause of PEMS [5]. There are conflicts about the replication of reoviruses in the intestine of turkeys [5]. It is suggested that turkey reoviruses cause severe bursa atrophy in poults at a young age that probably lead to a permanent immunosuppression [5], which in turns cause enteric disease by stimulating the opportunistic microorganisms. Moreover, experimentally infect young poults with turkey reovirus induced subclinical tenosynovitis [84]. Sharafaldin et al. investigated the pathogenesis of turkey reoviruses. The virus was detected in cloacal swabs at 1–2 dpi and peaked at 14 dpi. Additionally, cytokines were elevated in intestines (at 7–14 dpi) and in gastrocnemius tendons (at 14 dpi), suggesting a possible correlation between viral replication and cytokine response in the early infection. Still, there is limited information about the pathogenesis of reovirus in turkeys and development of its diagnosis and control [85].

## 3. Diagnosis of Viruses Implicated in Turkey Poult Enteritis

The diagnosis of PEMS based on the clinical picture and gross lesions is difficult because there are no specific clinical signs or pathognomonic lesions. Generally, cultivation of some enteric viruses is also a challenge. Therefore, negative contrast electron microscopy (EM), PCR and serology can help in the diagnosis of viruses causing enteric complex in turkeys. The combined use of TEM and PCR in the diagnosis of PEMS is possible to potentiate the advantages of both methods. While the strengths of the TEM lie in the detection of the entire spectrum of virus groups, the PCR can be used for a more sensitive and differentiated diagnosis after narrowing down the spectrum to certain viruses.

The negative contrast EM enables the discovery and morphological assessment of all microorganisms present in a sample and thus offers great advantages compared to many other used methods, Although the visual representation of viruses using EM requires special knowledge regarding sample preparation and evaluation, this method enables the detection of the entire spectrum of viruses in just one preparation approach with reasonable effort. EM can be particularly successful in virus infections with a high presence of pathogens, for example during an acute infection phase [86]. Enteric viral infections stand out through the excretion of large amounts of pathogen in the feces and thus offer good requirements for the visual detection and differentiation of the virus particles based on specific morphological features [87,88]. In addition, with samples with a lower virus concentration, significant virus enrichment can be achieved by means of ultracentrifugation [89]. In addition to the identification of known viruses that can be recognized in the context of mono or mixed infections, it is also possible to discover new and previously unknown viruses [69,90]. The negative contrasting electron microscope is an ideal instrument for both individual bird diagnostics and for flock monitoring studies [87,91]. As a rule, 1–2% solutions of the heavy metal salts of molybdenum, tungsten or uranium are used as contrast media [92]. However, phosphotungstic acid (PTA) can be also used for contrasting because of its low toxicity [87].

Molecular-based methods are used also in the diagnosis of enteric viruses of turkeys [16,43,49,93,94,95,96,97,98]. Sellers and his colleagues developed a multiplex RT-PCR for simultaneous detection of enteric viruses in turkeys. Later on [79] developed a multiplex RT-PCR test for the detection and differentiation of turkey astrovirus-1 (TAstV-1), TAstV-2, ANV, chicken astrovirus (CAstV) and rotavirus in turkey and chicken samples [46]. El-Adawy and others developed and validated a simple, sensitive, specific and cost-effective multiplex PCR (mPCR) assay as a molecular screening approach for the detection of six enteric avian pathogens; *Campylobacter* spp., *Salmonella* spp, *Clostridium perfringens*, *Escherichia coli*, *Histomonas meleagridis* and *Tetratrichomonas gallinarum* for use in the daily practice of a clinical microbiology laboratory. The sensitivity and specificity of multiplex polymerase chain reaction (mPCR) was tested and evaluated. The mPCR is advantageous when compared with conventional detection methods because it allows detecting and distinguishing multiple pathogenic agents through the use of one test. It is cost effective, time saving, specific and sensitive [99].

**Figure 1:**
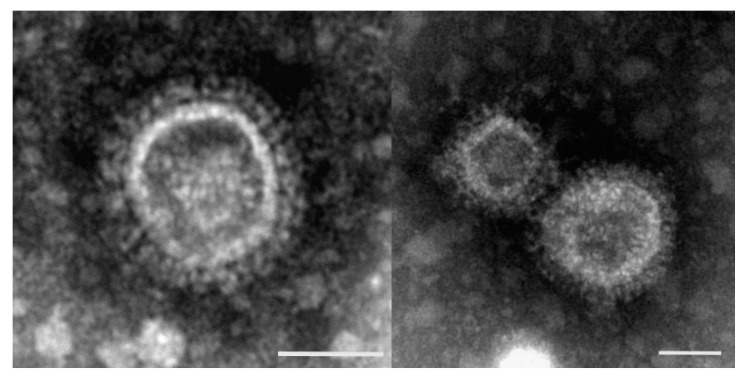
Corona-like particles (100–150 nm) in fecal samples of turkeys. Negative staining with phosphotungstic acid revealed that TCoVs are enveloped particles, roughly spherical, with diameters ranging from 100 to 200 nm (scale bar: 50 nm).

The diagnosis of TCoV depends on EM, PCR and serology. EM was used for identification of TCoV in turkey poults suffering from PEMS. TCoV was detected in turkey poults located in Germany using EM. When identifying TCoV particles, it must be considered that fragments of cell membranes often look very similar to these virus particles (Figure 1).

This often makes it difficult to clearly assess electron microscopic specimens. Immunoelectron microscopy can also help in these cases. However, a virus-specific antiserum is needed for this method of preparation. The detection of TCoV using PCR is described in several studies. Due to the great genetic homology between the IBV and TCoV [10,100], the first attempts to detect TCoV using PCR were based on the genetic analysis of already sequenced IBV strains [43,46]. A highly conserved non-coding region (3’UTR) at the 3’-end of the RNA strand is particularly suitable for the design of PCR primers [101,102]. The 3’UTR sequence fragments showed high homologies between the TCoVs strains and IBV [103]. Based on this fact, IBV (e.g., IBV vaccine strain H120) can be used as a positive control for the identification of TCoV based on 3’UTR-PCR [20,23]. Additionally, based on the fact that N and M genes are highly conserved, there were attempts to identify TCoV using PCR targeting these genes [43,46,103]. The high sensitivity of this diagnostic method makes it possible to detect even very small amounts of virus particles. It was found that TCoV can be detected in cloacal swabs just 24 h after an oral infection of turkey poults [5,42]. The use of the 3’UTR primers and the N-gene primers had identical results. Despite the fact that no coronavirus genome was found in the of Bursa Fabricii samples, detection was successful in at least 27% of the cloacal swabs. These results highlighting that feces and intestinal samples are the best samples suited for PCR detection of TCoV [22].

The ELISA and immunofluorescent assay (IFA) can be used for the detection of antibodies in sera collected from birds at 10–15 days after the onset of clinical signs to help in the diagnosis of PEMS. Commercially available ELISA plates that are coated with IBV antigens could be successfully used for the detection of antibodies to TCoV in antibody-capture ELISA [104]. The recombinant S1 spike polypeptide was also used to develop a TCoV-specific antibody ELISA [42]. Abdelwahab and others developed a recombinant ELISA based on the N protein of TCoV expressed in a prokaryotic system for the detection of antibody of TCoV. The relative sensitivity and specificity of the recombinant ELISA compared with IFA were 86% and 96%, respectively [105].

In contrast, TCoV could not be detected in turkey stocks suffering from PEMS [50,51], highlighting the fact that PEMS is a multifactorial syndrome and other potential causative agents should also be investigated.

**Figure 2:**
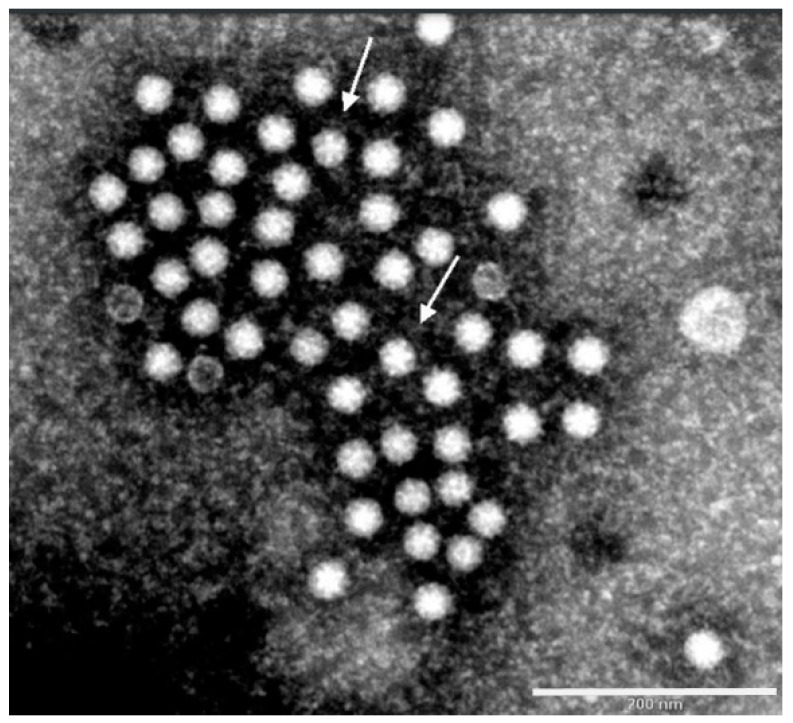
Astrovirus particles using electron microscopy (CVUA-Stuttgart 2010), showing five or six-rayed star-shaped particles (white arrow) using negative contrast EM (scale bar: 200 nm).

The diagnosis of astroviruses can be done based on EM and RT-PCR. The TAstV was recognized as five or six-rayed star-shaped particles by negative contrast EM from samples collected from poults suffering from PEMS in Germany (Figure 2). However, this morphology only applies to about 10% of all the particles shown, while the rest have a smooth surface [4,58,106,107]. In some cases, it is difficult to distinguish between astrovirus particles and other enteral viruses such as picorna and enteroviruses, so that such particles are often referred to as small round viruses (SRV) [58]. The average size of the particles is 29.6 nm [106]. The RT-PCR can be used also for the diagnosis of TAstV using primers specific to the polymerase or capsid genes [48,49,108]. Mixed infections with TAstV and TCoV have been reported in turkeys causing a severe negative impact on intestinal absorptive functions as causative factors of PEMS [55]. Additionally, coinfection of turkeys with TAstV and rotavirus was also reported in the US [65].

**Figure 3:**
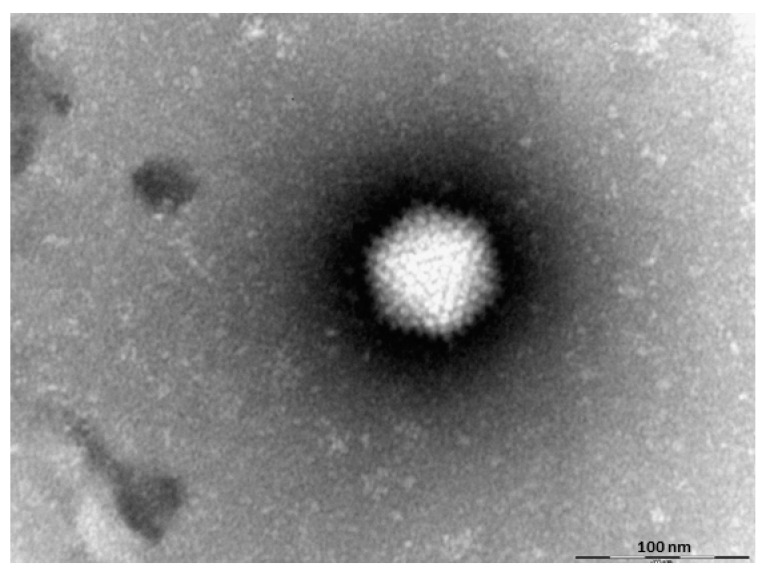
Adenovirus particles using electron microscopy (scale bar: 100 nm, CVUA-Stuttgart 2010).

The diagnosis of adenoviruses depends on virus isolation and molecular identification. Virus isolation can be done using cell cultures derived from the homologous species [94]. However, adenoviruses could be successfully derived from turkeys using chicken embryo liver (CEL) cells isolated from SPF chickens. The main cytopathic effects on CEL cells are rounded cell degeneration after the 1st and 4th passages [65]. Adenovirus could be also detected in turkey poults using EM (Figure 3). Molecular typing can be done based conventional PCR targeting the L1 region of the hexon gene [65,109]. Amplification and sequence analysis of the polymerase gene can be used to distinguish between TAdV-B, TAdV-C and TAdV-D [65,110].

**Figure 4:**
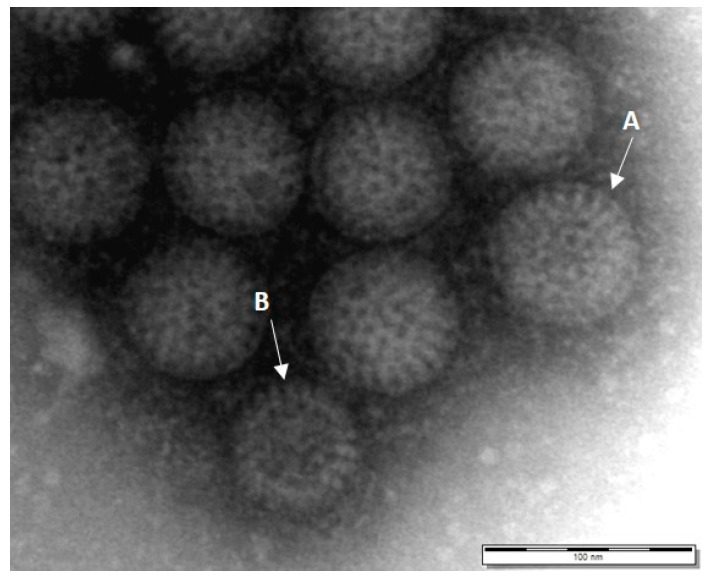
Rotavirus particles using electron microscopy. (**A**) intact virus particles; (**B**) Virus particles without an outer protein layer (scale bar: 100 nm, image, CVUA-Stuttgart).

Due to the high sensitivity of PCR in determining the genotype of rotaviruses, it is a good alternative to EM or virus antigen ELISA [111]. In human medicine, PCR methods are established that amplify sections of gene 4 (VP4), gene 9 (VP7) or gene 9 (NSP4) [108,112,113]. To detect avian rotaviruses using PCR, highly conserved primers of the NSP4 gene can be used [51,79]. The NSP4 gene sequences of the rotaviruses detected by these authors were 96.1%–97.5% identical. Rotavirus particles could also be detected in turkey poults using EM (Figure 4).

## 4. Prevention and Control of Enteritis in Turkeys

PEMS is an interaction between enteric pathogens and opportunistic infections in young turkeys. The main role of enteric viruses as primary agents in this syndrome is not fully understood. However, it is obvious that the interaction between enteric viruses and opportunistic bacteria/parasites and management increased the pathological effects. The development of PEMS depends on the virus–host interaction, virus pathotype, age of birds, immune status, biosecurity, and healthy conditions of the GIT. Therefore, several measures should be taken to control PEMS in turkeys, (1) reduction of the pathogenic load using antibiotic alternatives, (2) maintaining gut healthy and strengthening the immune system, (3) hygienic measures and (4) vaccinations.

There is an increasing trend to use alternatives to antibiotics including probiotics, prebiotics, organic acids, essential oils and botanical extracts for turkey [114] in the aim of reducing the pathogenic load. Several studies highlighted the benefits of these products in the improvement of animal performance and reduction infections in turkeys [115,116,117,118,119,120]. Lactic acid bacteria (LAB) proved to be an efficient antibiotic alternative to control *Salmonella* in turkeys by the reduction of intestinal colonization of *Salmonella* Typhimurium [121] and *Salmonella* Enteritidis [122]. Higgins and others found also that supplementation of turkey poults with LAB following antibiotic treatment improved significantly animal performance, compared with non-treated or probiotic-treated poults [123]. Additionally, Leyva-Diaz found that combinations between curcumin and copper acetate reduced the colonization of *Salmonella* Typhimurium in turkey poults and maintained a better intestinal homeostasis [124]. It was also found that *Propionibacterium freudenreichii* subsp. *freudenreichii* modulated the beneficial microbiota and reduced the multidrug-resistant *Salmonella* Heidelberg colonization in turkey poults [125].

Several medical plants such as rosemary, sage, thyme and oregano exhibited a broad-spectrum of antimicrobial properties and antioxidative effects [126,127] and improved animal performance in turkeys [128]. Essential oils have also a broad spectrum of antibacterial and antiparasitic effects [129,130] by increasing the permeability of the cell wall of microorganisms and/or inactivation cellular enzymes [130]. It was found that benzoic acid and essential oils improved performance, increased lactic acid bacteria populations and decreased coliform bacteria in the caeca of turkey poults [131]. Additionally, thymol and essential oils improved the antioxidant status of turkeys. Similar results were described in broiler chickens in which essential oils improved the intestinal microbial balance through reduction of coliform bacteria and increasing the *Lactobacillus* spp. of commercial broiler chickens [132].

Generally, protozoan and immunosuppressive diseases such as Marek’s disease in turkeys should be taken in to consideration [133]. Improvement of the immune system of turkey poults has a role to resist diseases. The β-glucans have an immunomodulatory effect due to increasing the activity of immune cells such as macrophages and neutrophils [134,135,136]. It also decreases *Salmonella* Enteritidis invasion and stimulates phagocytosis, bacterial killing, and oxidative burst in heterophils isolated from 4-d-old male Leghorn chickens 24 h after the oral challenge [137]. Supplementation of turkeys with probiotics (mannan-oligosaccharide) in combination with probiotics enhanced the immunoglobulin levels and improved performance [138]. Due to the negative impacts of protozoan such as *Histomonas meleagridis* in turkeys on the health and welfare, preventative management measures should be strictly applied to prevent the infection [2]. Additionally, litter management requires also thoughtful consideration and active management [139].

Although there is no specific treatment, the prevention of PEMS includes vaccination against potential pathogens in the case of available vaccines and hygienic measures. Until now, there are no available vaccines against viruses causing PEMS. In addition, there is no specific treatment. Although several efforts were done to develop effective vaccine against TCoV using classical methods (attenuated and inactivated vaccines) and molecular based (DNA and vector) vaccines, early and protective humoral and cellular immune responses could not be obtained by the developed vaccines [25]. Further improvements and optimization of vaccination regimes against TCoV are urgently needed.

## 5. Conclusions and Recommendations

Although no etiological agent has been identified as a specific cause of PEMS condition, several potential infectious agents and non-infectious predisposing factors are associated with this condition. The fact that the pathogenicity of several enteric viruses in turkeys remains unclear and it cannot be excluded that PEMS initiated by an unidentified virus. Opportunistic microorganisms such as *Salmonella*, *E. coli*, *Clostridium* and parasitic infections complicate the disease, leading to severe economic losses. Although isolation of the enteric viral agents is a challenge, EM and molecular identification can be used for diagnosis. No vaccine against viruses associated with this condition are available. Biosecurity including disposal of dead birds, litter management is very important in preventing the spread of any infectious agent between farms and between birds of different ages within the farm. Additionally, general management measures such as raising the temperature, use of antibiotic alternatives to combat secondary bacterial infections and supportive treatment might minimize the economic losses. Maintaining healthy gut and strengthening the immune system of turkey poults are crucial factors to prevent enteritis in turkeys. This can be achieved by supplementation of birds with probiotics, prebiotics and/or phytogenic substances.

## Figures and Tables

**Table 1:** Possible causes of enteric disorders in turkeys.

**Infectious**	**Viral**	Newcastle disease virus (*Paramyxoviridae*) Avian influenza A (*Orthomyxoviridae*) Infectious bursal disease virus (*Birnaviridae*) Hemorrhagic enteritis virus (*Adenoviridae*) Coronavirus enteritis (*Coronaviridae*) Rotavirus (*Reoviridae*) Reovirus (*Orthoreoviridae*) Astrovirus (*Astroviridiae*) Enterovirus (*Picornaviridae*) Parvovirus (*Parvoviridae*)
	**Bacterial**	*Salmonella* spp. *E. coli* *Clostridia* spp. *Chlamydia* spp.
	**Mycotic**	*Candida*
	**Parasitic**	*Ascaridia* *Coccidia*
**Non-infectious**	**Nutritional**	Feed structure, palatability, energy content and pellet quality
	**Management**	Temperature, stocking density, available feed space, available water space, distribution of feeders and air quality

## Data Availability

Data is contained within the article.
